# Dr. Charles Lisle Bond

**Published:** 1911-04

**Authors:** 


Dr. Charles Lisle Bond, University of Buffalo (1903) died from pneumonia at his home in Vulois, N. Y. February 25, 1911. aged 38 years.

